# Early-life vancomycin treatment promotes airway inflammation and impairs microbiome homeostasis

**DOI:** 10.18632/aging.101901

**Published:** 2019-04-13

**Authors:** Xin Yang, Hanrong Feng, Xueqin Zhan, Chao Zhang, Rui Cui, Lijia Zhong, Songmin Ying, Zhimin Chen

**Affiliations:** 1 Department of Pulmonology, Children’s Hospital, Zhejiang University School of Medicine, Hangzhou, China; 2 Department of Pharmacology and Key Laboratory of Respiratory Disease of Zhejiang Province, Department of Respiratory and Critical Care Medicine, Second Affiliated Hospital, Institute of Respiratory Diseases, Zhejiang University School of Medicine, Hangzhou, China; 3 Department of Anatomy and Cell Biology, School of Medicine, Zhejiang University, Hangzhou, China

**Keywords:** vancomycin, asthma, eosinophil, allergic airway inflammation, microbiome

## Abstract

Several studies have reported that gut and lung microbiomes are involved in the process of asthma pathogenesis. However, it remains unclear how perinatal or early-life antibiotic intervention affect adult allergic airway inflammation. We assigned C57BL/6 mice randomly to four experimental groups: normal saline control (NS), ovalbumin (OVA), vancomycin pretreated NS (VAN-NS), and vancomycin pretreated OVA (VAN-OVA). The vancomycin groups were orally given the drug from gestational day 14 to 6 week. An OVA-induced asthma model was then established at 6 weeks of age, and airway inflammation was evaluated. In addition, total DNA was extracted from the feces and lung tissue and used for 16S rDNA gene sequencing, to detect the composition of the microbiome. In the VAN-OVA group, airway inflammation and Th2-related cytokines were found to be significantly increased versus the control groups. Gene sequencing showed that vancomycin treatment attenuated the richness and evenness, and altered the composition of the microbiome in the gut and lung. *Micrococcaceae* and *Clostridiaceae-1* were potentially correlated to the severity of allergic airway inflammation. Our study suggests that perinatal and early-life vancomycin intervention aggravates allergic inflammation in adulthood, which might be correlated with imbalanced gut and lung microbiome homeostasis.

## Introduction

Asthma is defined as an allergen-induced chronic airway inflammation disease, characteristic of airway hyperresponsiveness (AHR), reversible airflow restriction, and airway remodeling. The clinical symptoms include coughing, wheezing, dyspnea, and chest tightness. Asthma is thought to be caused by a combination of genetic and environmental factors [1]. More recently, several internal factors in the microbiome have also been shown to play an important role in the pathogenesis of asthma [2]. Whether infections or exposure to microorganisms, the changes in the host microbiome composition are related to asthma development [3,4]. Personal hygiene improvement and declining family size reduce early-life microbiome exposure are associated with an increased risk of atopic diseases [5].

The last century has shown an alarming rise in immune dysregulation diseases, leading to the hygiene hypothesis. The prevalence of these immune disorders is thought to be associated with reduced type 1 immune responses. In the past few decades, several studies have demonstrated that microbial dysbiosis is important for immune imbalance—especially the microbial colonization and immunostimulatory signals during early life or those passed on by the mother [6].

The occurrence and development of infectious diseases, obesity, diabetes, liver diseases, coronary heart disease, tumors, and hypersensitivity disease are closely related to the microbiome dysbiosis [7–11]. Many different bacteria colonize different parts of the body. For example, there are over 1,000 different bacterial species that encode about 5 million genes that colonize the intestinal tract. Moreover, each site is characteristic to the specific microbiota populations [12]. The intestinal tract is reported to be sterile *in utero*; gut microbiota colonization occurs after birth [13]. In the early stages of life, the composition of the intestinal microbiota is influenced by diet and changes with age [14]. The dominant phyla are *Firmicutes* and *Bacteroidetes* in the intestinal microbiome of healthy humans; there are also some *Proteobacteria*, *Actinobacteria*, *Verrucomicrobia*, and *Cyanobacteria* [15,16].

It has also been reported that neonatal and continuous vancomycin administration in the murine allergic asthma model increases major indicators of airway inflammation [17]. In contrast, our study was designed to treat the perinatal and early-life period in mice up to 6-weeks old, which is similar to the general clinical picture. We found that mice receiving vancomycin in perinatal and early life had irritated airway inflammation when they became adults. With 16S rDNA Amplicon Sequencing technology, we demonstrated that the microecology of the gut and airway changed after vancomycin treatment—which might correlate to airway allergic eosinophilia.

## RESULTS

### Perinatal and early-life vancomycin treatment aggravate airway inflammation in adulthood

**Figure 1:**
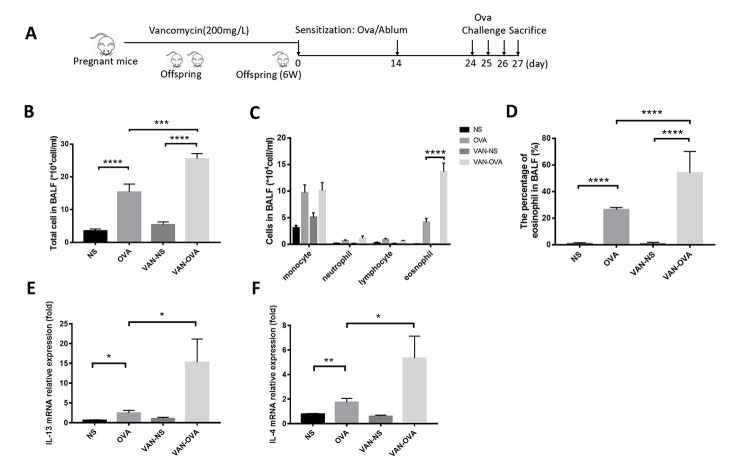
**Vancomycin use in perinatal and early life exacerbated allergic airway inflammation.** (**A**) Representative time course for the vancomycin procedure involved the murine asthma model. (**B, C**) Total cell count and cell differentiation of inflammation cells in BALF. (**D**) The percentage of eosinophils in BALF quantified by Wright-Giemsa staining (Radnor, PA, USA). (**E, F**) IL-13 and IL-4 relative mRNA expression in lung tissue was measured by Q-PCR. Data are shown as means ± SEM with 10 samples per group. (*P < 0.05; **P < 0.01; ***P < 0.001; ****P < 0.0001).

To clarify the effect of antibiotics in early life on asthma, we established the following models: mice being exposed to vancomycin from late pregnancy (gestational day 14) to 6 weeks of age, when an OVA-induced asthma model was established (Figure 1A). Compared to the OVA group, vancomycin administration led to an increasing tendency in total cell number and eosinophils (absolute counts and differential) in bronchoalveolar lavage fluid (BALF) (Figure 1B–1D). The inflammatory cytokines IL-13 and IL-4 from lung tissue were elevated and compared to mice without vancomycin administration by quantitative real-time PCR (Q-PCR) (Figure 1E, 1F).

**Figure 2:**
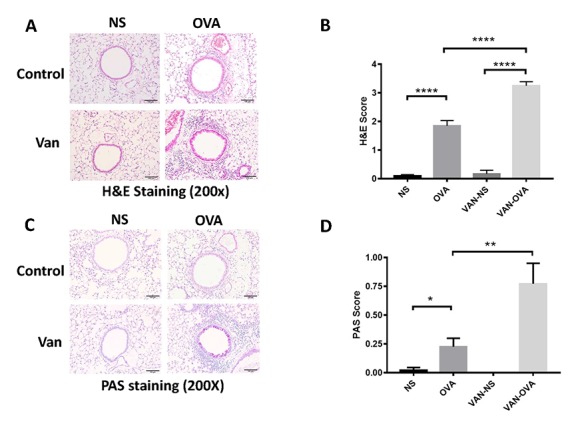
**Vancomycin aggravates inflammatory cell infiltration and mucus secretion in the OVA-induced asthma model.** (**A, C**) Representative H&E and PAS staining of lung sections. (**B, D**) Peribronchiolar and perivascular inflammation score, and mucus production with semi-quantification (score: 0–4) under the microscope (n = 8) (10 × 20 magnification). Data shown as means ± SEM. (*P < 0.05; **P < 0.01; ***P< 0.001; ****P< 0.0001).

We then examined the pulmonary pathology sections stained with hematoxylin/eosin (H&E) and periodic acid-Schiff (PAS) staining. In the OVA-induced asthmatic mice, inflammatory cells around the trachea and mucin secretion markedly increased in comparison to the control group. In the vancomycin-treated group, both the inflammation cells and mucus production were significantly increased versus the OVA group (Figure 2).

### Vancomycin alters the richness and evenness of the gut and lung microbiome

To further investigate how vancomycin affects OVA-induced airway inflammation, we collected the feces and lung tissue of the mice, and examined the composition of the microbiome via 16S rDNA gene sequencing. The differences in the species and quantity of microbiota were obvious between these groups.

**Figure 3:**
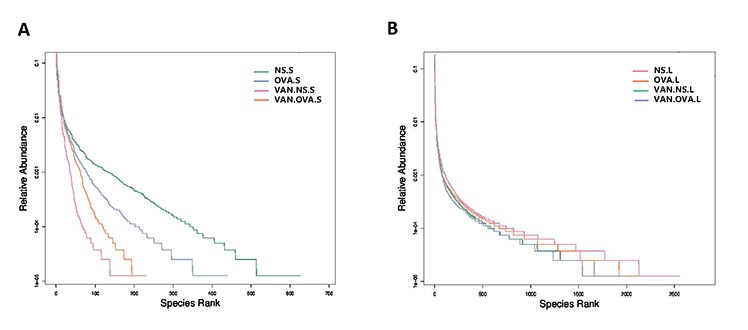
**Vancomycin use reduced the richness and evenness of the microorganisms in the gut and lung.** Rank-Abundance curves of all experimental groups. Lateral axis indicates the richness of samples, slope of curves indicates evenness of the microbiome in the (**A**) gut and (**B**) lung (n = 3). (NS.S: stool sample of the NS group; OVA.S: stool sample of the OVA group; VAN.NS.S: stool sample of the VAN-NS group; VAN.OVA.S: stool sample of the VAN-OVA group; NS.L: lung tissue of the NS group; OVA.L: lung tissue of the OVA group; VAN.NS.L: lung tissue of the VAN-NS group; VAN.OVA.L: lung tissue of the VAN-OVA group).

The rank-abundance curve describes the richness and evenness of the microbial species and showed that these difference between groups were insignificant. In the VAN-NS and VAN-OVA groups, the richness and evenness of the microbiota was reduced in the gut and the lung, compared to the NS and OVA groups (Figure 3A, 3B). This suggests that vancomycin administration impaired homeostasis and reduced these variables in the gut and lung microbiota.

### Vancomycin changes the composition of the gut and lung microbiome

**Figure 4:**
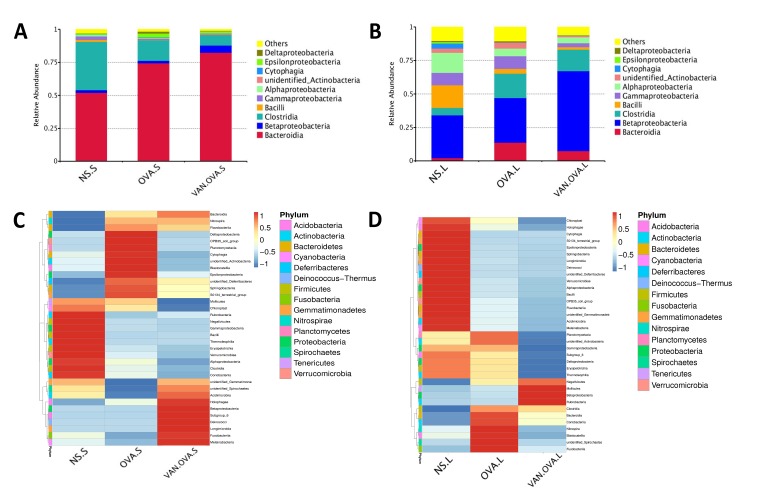
**Vancomycin pretreatment alter the microbiome in both gut and lung in the OVA-induced asthma model.** (**A, B**) Composition of bacterial community at class level. The top 10 species are shown, and the other phyla are included as “Others”. Relative abundance of the (**A**) gut and (**B**) lung microbiota at the class level. (**C, D**) Heatmap showing the relative abundance of the top 35 bacterial genera in the gut and lung microbiome, depicted by color intensity. The relative abundance at the class level of the (**C**) gut and (**D**) lung microbiota (n = 3) (NS.S: stool sample of the NS group; OVA.S: stool sample of the OVA group; VAN.OVA.S: stool sample of the Van-OVA group; NS.L: lung tissue of the NS group; OVA.L: lung tissue of the OVA group; VAN.OVA.L: lung tissue of the Van-OVA group).

We analyzed the top ten species in each group at the class level—and found that the proportion of dominant bacteria varied in different groups. In feces samples of the NS group, the top three species were *Bacteroidia* (51.97%, which belong to phylum *Bacteroidetes*), *Clostridia* (36.53%, which belong to phylum *Firmicutes*), and *Betaproteobacteria* (2.03%, which belong to phylum *Proteobacteria*). In the feces of OVA-induced allergic asthma mice, the abundance of *Clostridia* distinctly decreased to 15.48%, and the *Bacteroidia* increased to 74.35%. In the VAN-OVA group, *Clostridia* decreased from 15.48% to 8.14%, while *Bacteroidia* was enriched to 82.45% from 74.35%; *Betaproteobacteria* was enriched to 5.3% from 1.9%, versus the OVA group (Figure 4A).

The three dominant genera in the lung tissue of the NS group were *Betaproteobacteria* (31.97%, belonging to phylum *Proteobacteria*), Bacilli (16.79%, belonging to phylum *Firmicutes*), and *Alphaproteobacteria* (14.81%, belonging to phylum *Proteobacteria*). In the OVA group, the proportion of *Bacilli* decreased from 16.79% to 3.6%, while *Alphaproteobacteria* decreased from 14.81% to 5.6% compared to the NS group. This tendency to decrease was heightened with vancomycin treatment. In the VAN-OVA group, the proportion of *Bacilli* decreased to 1.88%, and *Alphaproteobacteria* was 4.42% in the lung microbiome. There are many bacterial genera that increased with vancomycin intervention: *Betaproteobacteria* was enriched to 59.86%, while the proportion was 33.44% in the OVA-induced asthma group (Figure 4B).

We created a hierarchical cluster analysis of bacterial communities at the class level to demonstrate their different composition more clearly. The result showed that the relative abundance of the top 35 bacterial genera in the groups were significantly different. This also suggested that the microbiota in local sites are unique under different pathological conditions (Figure 4C, 4D, and Supplementary Figure 1A, 1B).

### Perinatal and early-life vancomycin treatment alters microbiome abundance in the gut and lung of an OVA-induced asthma model

**Figure 5:**
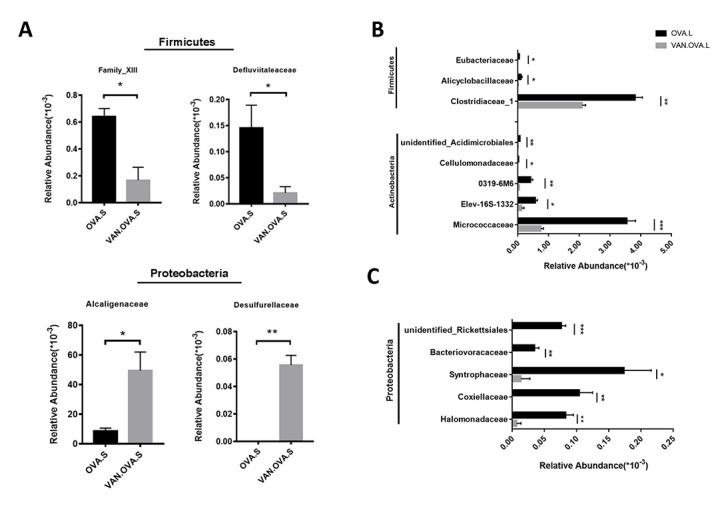
**The representatively changed bacteria in the gut and lung followed by vancomycin administration in the OVA-induced asthma model.** (**A**) Relative abundance of *Family_XIII*, *Defluviitaleaceae*, *Alcaligenaceae* and *Desulfurellaceae*. Different symbols represent the fecal samples from each group. (**B, C**) Changed species in the lung were classified into 3 groups. The representative bacteria were listed based on the relative abundance. Data were shown as means ± SEM, n = 3. (*P< 0.05. **P < 0.01; ***P < 0.001) (OVA.S: stool sample of the OVA group; VAN.OVA.S: stool sample of the Van-OVA group; OVA.L: lung tissue of the OVA group; VAN.OVA.L: lung tissue of the Van-OVA group).

The vancomycin intervention markedly changed the composition of the gut microbiome. At the family level, two defined *Firmicutes* species, *Family_XIII* and *Defluviitaleaceae*, were detected having less abundance in the VAN-OVA group compared to the OVA group. In contrast, *Alcaligenaceae* and *Desulfurellaceae*, which belonged to phylum *Proteobacteria*, were detected at a higher abundance in the VAN-OVA group (Figure 5A). Moreover, there were significant changes in the lung microbiota at the family level in the VAN-OVA group versus the OVA group. Among the modified bacteria, the most obvious were: *Micrococcaceae*, *Halomonadaceae*, *Clostridiaceae-1*, *Elev-16s-1332*, *Coxiellaceae*, *Alicyclobacillaceae*, *0319-6M6*, *Cellulomonadaceae*, *Syntrophaceae*, *Bacteriovoracaceae*, *Rickettsiales*, *Eubacteriaceae*, and *Acidimicrobiales*. These bacteria were in phylum *Proteobacteria, Actinobacteria*, and *Firmicutes* (Figure 5B, 5C). Among the changed bacteria, *Micrococcaceae* and *Clostridiaceae-1* were more abundant than others in the airway; they decreased significantly with vancomycin intervention (Figure 5B). These local microbiotas were likely the most potentially correlated with the severity of allergic airway inflammation.

## DISCUSSION

The composition of the microbiome is believed to critically affect the host: its homeostasis can be disrupted by a variety of stimuli, including diet, illness, hormonal cycles, and medication. Accumulating evidence suggests that changes in the gut microbiome are important risk factors that induce abnormal tissue function and diseases, such as inflammatory bowel disease [18], obesity, insulin resistance [19,20], cardiovascular disease [21–23], allergic diseases, and even the brain’s cognitive function. Nevertheless, the first few months of life are a ‘critical window’, during which the infant’s gut microbiota might impact the immune system [24]. External interventions during perinatal and early life have been associated with the development of allergic diseases in adulthood [25].

Antibiotics are used widely in the treatment and prevention of bacterial infections [26], but their misuse can disrupt normal flora [27]. Antibiotics are linked to *Clostridium difficile*-associated diarrhea [28,29] and can increase the risk of obesity and type 1 diabetes in adulthood [30,31].

A previous study reported that vancomycin treatment in breeding pairs, lasting for the duration, caused an exacerbated allergic response and changes in intestinal flora in an OVA-induced murine asthma model [17]. This indicated that the use of vancomycin may be correlated to the pathological progress of asthma. Compared with, and modified as a function of this study, we focused on this intervention from the perinatal period to the early-life stage, and found that it exacerbated allergic airway inflammation in the subsequent OVA-induced asthma model. Our results verify that the early-life microbiome plays a critical role in the maturity of the host’s immune system, and that changes in the microbiome will have long-standing consequences [32]. In addition, we used 16S rDNA gene sequencing to study the impact of vancomycin exposure during perinatal and early life on the gut and lung microbiome. The richness and evenness of the microbial species in the gut and airway decreased after vancomycin intervention (Figure 3); the composition of the microbiome changed with vancomycin treatment. Alterations in the proportion of dominant microbiota might be associated with aggravated airway inflammation due to vancomycin intervention (Figure 4). We found that with the antibacterial effect of the vancomycin, the relative abundance of *Firmicutes* decreased in the gut, but the relative abundance of *Proteobacteria* increased, which varied from the previous report [17]. Thus, our results suggest that *Micrococcaceae* and *Clostridiaceae-1* correlated most strongly with the severity of allergic airway inflammation (Figure 5B).

However, whether changes in the airway microbiome cause asthma remain unclear. Studies have demonstrated that infants with *Streptococcus pneumonia*, *Haemophilus influenzae*, and *M. catarrhalis* in their oropharynx are more likely to suffer from asthma in adulthood [33]. This study used vancomycin in perinatal and early life to alter the microbiome before OVA stimulation. In later allergic airway inflammation, the data show significant irritation versus OVA-induced asthma. Therefore, we show that the perinatal and early-life period were “critical windows” during which the microbiota could influence the rate and pattern of maturation in immune function, as well as determine the severity of asthma in later life.

The gut microbes influenced lung disease through the gut-lung axis and the common mucosal immune system; however, this mechanism still remains unclear. Studies have shown that the gut microbiota releases some metabolites such as short-chain fatty acids, that will alter the local microbiome in the gut and make it less hospitable for colonization or overgrowth of pathogenic species [34]. This imbalance of the microbiome influences the T-regulatory cells, Th17 cells [35,36], and other immune-related cytokines leading to immune-related diseases.

As stated, our results suggest that vancomycin administered perinatally and in early life alter components of the gut microbiome and promote the release of Th2 cytokines. Vancomycin treatment also altered the lung microbiome that aggravates airway inflammation via the gut-lung axis or the common mucosal immune system. However, further studies are needed to investigate the mechanism between the airway microbiome and asthma. In this vein, several bacteria changed with vancomycin administration, which could be a new approach to asthma prevention and treatment.

## METHODS

### Mice

C57BL/6 mice (wild-type, aged 6 to 8 weeks) were purchased from the Slac Laboratory Animal Co. Ltd. (Shanghai, China). Breeders were mating them, and their offspring were selected for the following experiments: all mice were maintained in a specific-pathogen-free facility and given a standard diet with normal drinking water. The room temperature was maintained at 23 ± 2° C with 50% ± 10% humidity and a 12-h light/12-h dark cycle. The study was conducted in agreement with the Experimental Animal Welfare and Ethics Committee of Zhejiang University.

### Vancomycin treatment

C57BL/6 pregnant mice were randomly assigned to normal drinking water, with vancomycin-treatment groups assigned on day 14 of gestation. Mice in the vancomycin-treatment group were given vancomycin at 200 mg/L in drinking water from gestational day 14 to 6-weeks old. The offspring of control and vancomycin-treatment mice were randomly divided into normal saline (NS, n = 10) and OVA (n = 10) groups, respectively. Mice in the non-vancomycin-treatment group had drinking water for the entire experiment.

### OVA-induced allergic airway inflammation in the mouse model

The OVA-induced asthma model was established in mice at 6-weeks old. Mice in the OVA and VAN-OVA groups for the asthma model were sensitized on day 0 and 14 by intraperitoneal injection of 80 ug OVA (Sigma-Aldrich, St Louis, MO, USA) in 0.1 ml and an equal amount of aluminum hydroxide (Pierce, Rockford, IL, USA). The NS groups were given NS instead. On days 24 to 26, mice were challenged with 1.5% OVA for 35 min. Control mice in the NS and VAN-NS groups were exposed to saline aerosols. Parameters were analyzed at 24 h after the OVA challenge.

### BALF collection and differential cell count

At 24 h after final OVA challenge, mice were anesthetized and sacrificed for airway inflammation analysis. BALF was extracted from the left lungs with 0.4 ml PBS three times for a total volume of 1 ml. Total BALF cells were counted under a microscope. The remaining cells were centrifuged, spun onto glass slides, and stained with Wright-Giemsa staining buffer according to the manufacturer’s instructions; the number of differential cells were counted and classified under a microscope by finding > 200 cells.

### Histologic analysis

The left lungs were fixed in formalin for 24 h and then embedded in paraffin. The sections were stained with H&E and PAS. The inflammation was evaluated as described with the H&E staining sections [37]. The PAS score was assessed according to the former papers [38,39]. All slides were examined in a random blinded fashion by two independent investigators.

### RNA isolation and quantitative real-time PCR analysis

The lung tissue was lysed with Trizol reagent (Takara Biotechnology, Shiga, Japan). The cDNA was amplified with Reverse Transcription Reagents (Takara Biotechnology). The expression of IL13 and IL4 was measured by Q-PCR, using SYBR Green Master Mix (Takara Biotechnology, DRR041A) with a StepOne real-time PCR system (Applied Biosystems, Foster City, CA, USA). Primers used IL13 and IL4 Q-PCR as follows: *Il-13*: forward: 5′-CAGCCTCCCCGATACCAAAAT-3′, reverse: 5′-GCGAAACAGTTGCTTTGTGTAG-3′; *Il-4*: forward: 5’-CCCCAGCTAGTTGTCATCCTG-3’, reverse: 5’- CAAGTGATTTTTGTCGCATCCG-3’; *β-actin*: forward: 5′-GTCCACCGTGTATGCCTTCT-3′, reverse: 5′-CTCCTGGTGTCCGAACTGAT-3′.

### DNA purification from feces and lung tissue

The feces and lung tissue were cut in pieces with sterile scissors, and total DNA was extracted with QIAamp DNA Mini Kit (Qiagen, Germany) according to manufacturer’s instructions. The DNA quality and concentration (purity ratio A260/A280) were measured with a NanoDrop ND-1000 spectrophotometer, and stored at -80° C for other experiments.

### Bacterial 16S rDNA gene amplification and high-throughput sequencing

The 16S rDNA genes of the V4 regions were amplified, using specific primer (F:5’-GTGCCAGCMGCCGCGGTAA-3’, R:5’-GGACTACHVGGGTWTCTAAT-3’) with the barcode. All PCR reactions used Phusion® High-Fidelity PCR Master Mix (New England Biolabs, Thermo Fisher Scientific, Waltham, MA, USA). We mixed the same volume of 1X loading buffer (which contained SYB green) with PCR products and performed electrophoresis on 2% agarose gel for detection. Samples with a bright main strip between 400-450 bp were chosen for further experiments. The PCR products were mixed in equal density ratios. A mixture of PCR products was then purified with Qiagen Gel Extraction Kit (Qiagen, Germany). Sequencing libraries were generated using TruSeq® DNA PCR-Free Sample Preparation Kit (Illumina, San Diego, CA, USA) following the manufacturer's recommendations with index codes added. The library quality was assessed on the Qubit@ 2.0 Fluorometer (Thermo Fisher Scientific) and the Agilent Bioanalyzer 2100 system (Athens, GA, USA). Finally, the library was sequenced on an IlluminaHiSeq2500 platform and 250 bp paired-end reads were generated.

### Statistical analyses

The statistical differences among groups were analyzed with one-way ANOVA, and P values were shown in related graphs. All data were expressed as the mean ± SEM. The analyses and graphs were performed with GraphPad Prism 7.0 software (GraphPad Software Inc., San Diego, CA, USA). The level of statistical significance was set at a p-value < 0.05.

## Supplementary material

Supplementary Figure
