# Supplementary material for: Early-life vancomycin treatment promotes airway inflammation and impairs microbiome homeostasis
Source: Aging (Albany NY). 2019 Apr 13;11(7):2071–81. doi: 10.18632/aging.101901 (PMC6503881; doi:10.18632/aging.101901)
Supplement: Supplementary Figure [file aging-11-101901-s001.pdf]

SUPPLEMENTARY MATERIAL

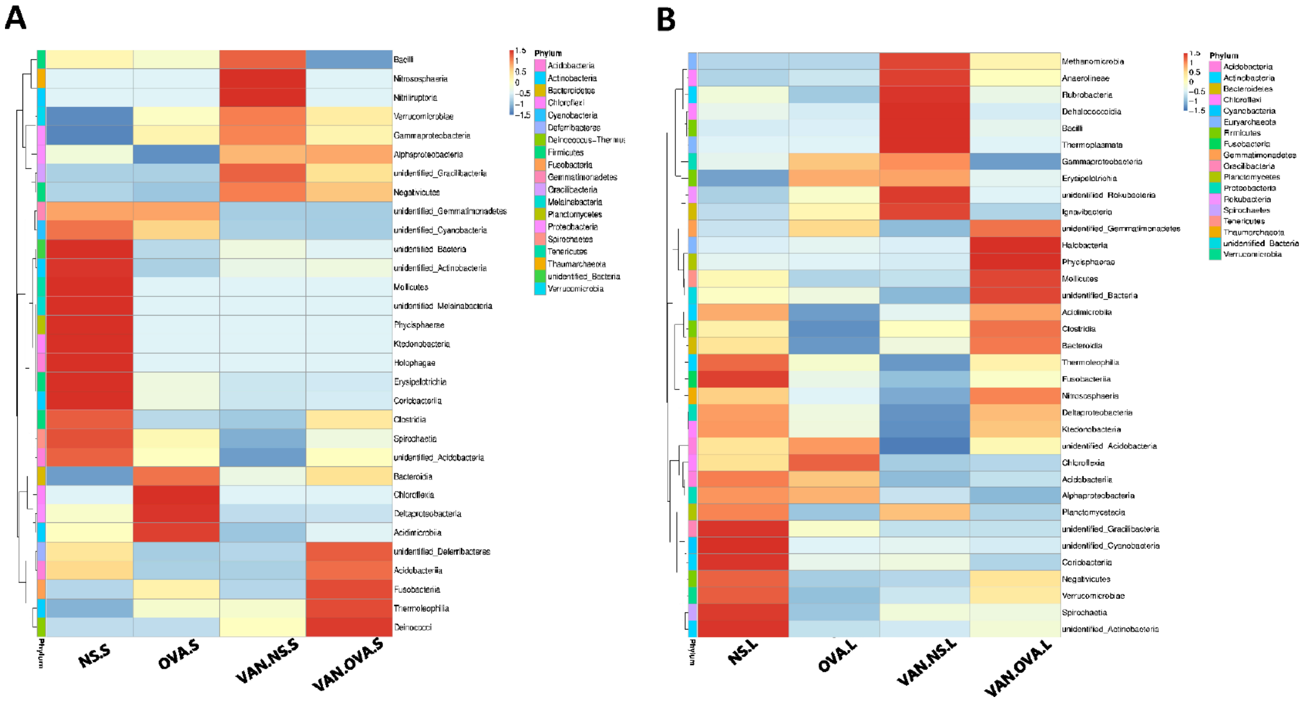

Supplementary Figure 1. Heatmap showing relative abundance of the top 35 bacterial genera in the gut and lung microbiome, depicted by color intensity. Relative abundance at the class level of the (**A**) gut and the (**B**) lung microbiota (n = 3) (NS.S: stool sample of the NS group; OVA.S: stool sample of the OVA group; VAN.NS.S: stool sample of the VAN-NS group; VAN.OVA.S: stool sample of the Van-OVA group; NS.L: lung tissue of the NS group; OVA.L: lung tissue of the OVA group; VAN.NS.L: lung tissue of the VAN-NS group; VAN.OVA.L: lung tissue of the Van-OVA group).
